# Predictive accuracy of systolic blood pressure to left ventricular end-diastolic pressure ratio versus TIMI score for short-term mortality after primary percutaneous coronary intervention

**DOI:** 10.34172/jcvtr.32933

**Published:** 2024-12-23

**Authors:** Rajesh Kumar, Naveed Ullah Khan, Ali Bin Naseer, Zille Huma, Kalsoom Chachar, Maryam Samad, Muhammad Ishaq, Abiha Urooj, Uroosa Safdar, Muhammad Rasool, Sohail Khan, Jawaid Akbar Sial, Tahir Saghir, Nadeem Qamar

**Affiliations:** National Institute of Cardiovascular Diseases (NICVD), Karachi, Pakistan

**Keywords:** STEMI, Primary PCI, Systolic blood pressure, Left ventricular end-diastolic pressure, Prognosis

## Abstract

**Introduction:**

Aim of this study was to evaluate the predictive performance of systolic blood pressure (SBP) to left ventricular end-diastolic pressure (LVEDP) ratio for the prediction of in-hospital and short-term mortality in a contemporary cohort of patients with ST-segment elevation myocardial infarction (STEMI) undergoing primary percutaneous coronary intervention (PCI) at a tertiary care cardiac center.

**Methods:**

This study included a consecutive series of patients diagnosed with STEMI who underwent primary PCI. The SBP/LVEDP ratio and TIMI (Thrombolysis in Myocardial Infarction) score were calculated, and their ability to predict in-hospital and short-term mortality was evaluated by analyzing the area under the curve (AUC) on the receiver operating characteristics (ROC) curve.

**Results:**

This study involved 977 patients, with 780 (79.8%) being male and a mean age of 55.6±11.5 years. Among them, 191 (19.5%) had an SBP/LVEDP≤5.4. The in-hospital mortality rate was 4.3% (42), and the short-term all-cause mortality rate after a mean follow-up of 5.9±2.4 months was 15% (140). Patients with SBP/LVEDP≤5.4 had higher in-hospital mortality rates (14.1% vs. 1.9%; *P*<0.001) and short-term mortality rates (35.1% vs. 9.8%; *P*<0.001) compared to those with SBP/LVEDP>5.4. The AUCs of SBP/LVEDP and TIMI for predicting in-hospital mortality were 0.766 [0.681-0.851] and 0.787 [0.713-0.861], respectively. For short-term mortality, the AUCs of SBP/LVEDP and TIMI were 0.731 [0.682-0.780] and 0.736 [0.690-0.782], respectively.

**Conclusion:**

In conclusion, SBP/LVEDP showed sufficiently high predictive power comparable to the TIMI risk score. SBP/LVEDP is a readily available ratio that can rapidly provide valuable prognostic information during primary PCI.

## Introduction

 Ischemic heart disease (IHD) stands as a predominant global health concern, accounting for a significant portion of morbidity and mortality worldwide. Among its manifestations, “ST-segment elevation myocardial infarction” (STEMI) emerges as a particularly critical and life-threatening subtype.^1^ Fortunately, advancements in medical interventions, notably “primary percutaneous coronary intervention” (PCI) alongside evidence-based pharmacological strategies and procedural techniques, have notably enhanced outcomes for STEMI patients.^1^ However, despite these advancements, a notable proportion of individuals continue to face adverse outcomes, ranging from 2.5% to 10% within 30 days post-procedure.^2-4^ Such outcomes encompass a spectrum of complications, including cardiogenic shock, heart failure, arrhythmias, ventricular remodeling, recurrent infarction, thromboembolic events, valvular dysfunction, and sudden cardiac death,^5^ with variabilities influenced by factors such as infarction extent, comorbidities, and timely medical intervention.^6,7^

 Identifying patients at heightened risk of adverse events is paramount in clinical practice,^8^ enabling proactive measures to mitigate post-procedural complications.^9^ Several clinical indices and scoring systems have been devised for risk stratification in STEMI patients, including widely recognized ones like the TIMI, PAMI, GRACE, and CADILLAC scores.^10-13^ However, their complex calculations, often necessitating online calculators, limit their practical utility, particularly in high-volume PCI centers.^14^ Consequently, simpler bedside and invasive indices have garnered attention, leveraging parameters such as heart rate (HR), systolic blood pressure (SBP), “left ventricular ejection fraction” (LVEF), and “left ventricular end-diastolic pressure” (LVEDP).^8,9,15-17^ Invasive hemodynamic measurements during the procedure offer theoretical advantages, providing a more precise assessment of left ventricular loading conditions and afterload, thus potentially offering superior predictive power for adverse outcomes.^14^ Notably, recent literature underscores the significance of a low SBP to LVEDP ratio as an indicator of short-term mortality following primary PCI,^14^ yet comprehensive data on its predictive performance remain scarce.

 Therefore, this study aims to assess the predictive efficacy of the SBP to LVEDP ratio for in-hospital and short-term mortality in a contemporary cohort of STEMI patients undergoing primary PCI at a tertiary care cardiac center in a developing country.

## Materials and Methods

###  Study Population

 This study included a consecutive series of patients who were diagnosed with STEMI and underwent primary PCI at the National Institute of Cardiovascular Diseases (NICVD) from August 2020 to July 2021. The study protocol was approved by the ethical review board of the institution in accordance with the Declaration of Helsinki. All participants provided their consent to participate in the study after receiving a comprehensive explanation of the study’s objectives and procedures.

###  Inclusion and exclusion criteria

 The study primarily included adult patients (aged ≥ 18 years), of any gender, who met the diagnosis criteria for STEMI as defined below. These patients were promptly transferred to a catheterization laboratory for primary PCI within 12 hours of symptom onset, except for patients in cardiogenic shock who underwent primary PCI regardless of symptom duration. Patients who did not provide consent and those requiring multi-vessel intervention during the initial procedure were excluded from the study. Diagnostic criteria for STEMI were; “history of typical chest pain for at least 20 minutes” and presenting ECG finding of “ST elevation in at least two contiguous leads > 2mm in men or > 1mm in women in leads V2 to V3 and/or > 1mm in other contiguous chest leads or limb leads”

###  Data collection

 We collected data on various aspects of the routine workup for STEMI at presentation, including demographic details, patient risk profile, and 12-lead electrocardiography (ECG) results. The information obtained included the patient’s age (in years), gender, total ischemic time (in minutes), vital signs at presentation (blood pressure in mmHg and heart rate in bpm), routine lab investigations such as random plasma glucose level (in mg/dL) and serum creatinine level (in mg/dL). Additionally, we recorded the patient’s Killip class, presence of arrhythmias, cardiac arrest, intubation status, and type of myocardial infarction.

 All primary PCI procedures followed the standard management protocol for STEMI patients. We also gathered data on procedure characteristics and angiographic findings, such as thrombus burden, infarct-related artery, and the number of involved vessels. Furthermore, we obtained information on hemodynamic parameters, specifically LVEDP and LVEF. The SBP to LVEDP ratio (SBP/LVEDP) was obtained. SBP and LVEDP were measured invasively at the start of procedure with the help of a 6F multipurpose catheter placed in aorta and left ventricle, respectively. The zero reference line was taken at the level of right atrium and calibrated as per standard protocol before each patient was studied.

 Additionally, the standard TIMI (Thrombolysis in Myocardial Infarction) score was also obtained as per the standard calculation criteria as comparator to the SBP/LVEDP.^18^ All the patients were followed up to six months and occurrence of major adverse cardiovascular events (MACE) were recorded which included all-cause mortality, recurrent myocardial infarction needing revascularization, unplanned hospitalization due to heart failure, and thromboembolic events such as stroke or cerebrovascular events (CVA). The last known status of patients was considered to mark the short-term MACE status of patients.

###  Data analysis

 Data collected for analysis were entered into IBM SPSS 21 software and analyzed accordingly. Receiver operating characteristic (ROC) curve analysis was conducted to evaluate the predictive performance of SBP/LVEDP and the TIMI score for in-hospital mortality. The optimal cutoff value was determined using maximum Youden’s J statistic. Based on this optimal cutoff value, patients were divided into two groups. Clinical and procedural characteristics, as well as in-hospital and short-term outcomes, were compared between these two groups using appropriate statistical tests.

 For variables that followed an approximately normal distribution, the independent sample t-test was used. If the variables were not normally distributed, the Mann-Whitney U test was employed. Categorical response variables were compared using the Chi-square test, and in cases where the expected cell frequency was low, Fisher’s Exact Test or Likelihood ratio test was applied as appropriate.

 The analysis provided the area under the curve (AUC), sensitivity (proportion of actual positives that are correctly identified), specificity (proportion of actual negatives that are correctly identified), accuracy (proportion of correct predictions among all predictions made), positive predictive value (proportion of actual positives among the samples that were predicted as positive), and negative predictive value (proportion of actual negatives among the samples that were predicted as negative), along with their corresponding 95% confidence intervals (CI). All statistical analyses were performed with a significance criterion of *p*-value < 0.05.

## Results

###  Baseline and clinical characteristics

 A total of 977 patients were included in this study, of whom 780 (79.8%) were male. The mean age of the patients was 55.6 ± 11.5 years, with 149 (15.3%) being under the age of 45. During presentation, 101 (11.4%) patients were classified as Killip class III/IV, 121 (12.4%) exhibited arrhythmias, 59 (6%) experienced cardiac arrest, and 130 (13.3%) required intubation. A ratio of SBP to LVEDP ≤ 5.4 was observed in 191 (19.5%) patients.

 Patients with SBP/LVEDP ≤ 5.4 were found to have longer ischemic time (median, 390 [290-540] vs. 330 [230-470]; *P* = 0.001), higher heart rate (mean, 91 ± 29.3 vs. 82.8 ± 16.7; *P* < 0.001), a higher incidence of anterior wall myocardial infarction (72.3% vs. 47.7%), a higher prevalence of Killip class III/IV (28.8% vs. 1.9% and 20.4% vs. 0.3%), a higher occurrence of arrhythmias and cardiac arrest at the time of presentation (32.5% vs. 7.5%; *P* < 0.001 and 20.9% vs. 2.4%; *P* < 0.001), and a higher prevalence of diabetes (49.2% vs. 36%; *P* = 0.001) compared to those with SBP/LVEDP > 5.4 (Table 1).

**Table 1:** Comparative distribution of clinical and demographic characteristics between the two study groups based on SBP to LVEDP ratio

**Characteristics**	**Total**	**SBP/LVEDP**		***P*** **-value**
		**≤5.4**	**>5.4**	
**Total (N)**	**977**	**191 (19.5%)**	**786 (80.5%)**	**-**
**Gender**				
Female	20.2% (197)	20.4% (39)	20.1% (158)	0.922
Male	79.8% (780)	79.6% (152)	79.9% (628)	
**Age (years)**	55.6 ± 11.5	57.2 ± 12.6	55.2 ± 11.1	0.048
< 45 years	15.3% (149)	14.1% (27)	15.5% (122)	0.042
45 to 64 years	59.9% (585)	53.9% (103)	61.3% (482)	
≥ 65 years	24.9% (243)	31.9% (61)	23.2% (182)	
**Total ischemic time (hours)**	348 [240-480]	390 [290-540]	330 [230-470]	0.001
**Heart rate (bpm)**	84.4 ± 20	91 ± 29.3	82.8 ± 16.7	< 0.001
**Systolic blood pressure (mmHg)**	130.9 ± 24.9	110.6 ± 23.2	135.8 ± 22.8	< 0.001
**Serum creatinine (mg/dL) on arrival**	1.0 ± 0.5	1.1 ± 0.4	1.0 ± 0.5	0.102
**Random glucose level (mg/dL)**	156 [130-209]	178 [130-238]	155 [129-200]	0.027
**Type of myocardial infarction (MI)**				
Anterior	52.5% (513)	72.3% (138)	47.7% (375)	< 0.001
Inferior	20.2% (197)	7.3% (14)	23.3% (183)	
Inferior with RV	18.3% (179)	13.6% (26)	19.5% (153)	
Inferio-posterior	5.5% (54)	5.8% (11)	5.5% (43)	
Lateral	1.8% (18)	0% (0)	2.3% (18)	
Posterior	1.6% (16)	1% (2)	1.8% (14)	
**Killip Class**				
I	77% (752)	34% (65)	87.4% (687)	< 0.001
II	11.7% (114)	16.8% (32)	10.4% (82)	
III	7.2% (70)	28.8% (55)	1.9% (15)	
IV	4.2% (41)	20.4% (39)	0.3% (2)	
**Intubated**	13.3% (130)	45% (86)	5.6% (44)	< 0.001
**Arrhythmias on presentation**	12.4% (121)	32.5% (62)	7.5% (59)	< 0.001
**Cardiac arrest**	6% (59)	20.9% (40)	2.4% (19)	< 0.001
**Co-morbid conditions**				
Hypertension	57.5% (562)	60.2% (115)	56.9% (447)	0.402
Diabetes mellitus	38.6% (377)	49.2% (94)	36% (283)	0.001
Smoking	30.9% (302)	24.6% (47)	32.4% (255)	0.036
Family history of IHD	1.9% (19)	1.6% (3)	2% (16)	> 0.999
Prior PCI	7.2% (70)	8.9% (17)	6.7% (53)	0.300
History of CVA/TIA	1.9% (19)	2.1% (4)	1.9% (15)	0.776

SBP = systolic blood pressure, LVEDP = left ventricular end-diastolic pressure, IHD = ischemic heart diseases, RV = right ventricular, PCI = percutaneous coronary intervention, TIA = transient ischemic attack, CVA = cerebrovascular accidents

###  Angiographic and procedural characteristics

 Patients with SBP/LVED *P* ≤ 5.4 were found to have higher need of IABP placement (19.9% vs. 0.9%; *P* < 0.001), three vessel involvement (41.4% vs. 28.4%), pre-procedure TIMI 0 flow (71.7% vs. 52.3%), high thrombus grade (G5, 70.7% vs. 51.4%), and low post-procedure TIMI III flow (76.4% vs. 92.6%) compared to those with SBP/LVEDP > 5.4 (Table 2).

**Table 2:** Comparative distribution of angiographic and procedural characteristics and in-hospital mortality between the two study groups based on SBP to LVEDP ratio

**Characteristics**	**Total**	**SBP/LVEDP**		***P*** **-value**
		**≤5.4**	**>5.4**	
**Total (N)**	**977**	**191 (19.5%)**	**786 (80.5%)**	**-**
**Left ventricular end-diastolic pressure (mmHg)**	40.9 ± 9.1	32.6 ± 8.1	42.9 ± 8.1	< 0.001
**Left ventricular ejection fraction (%)**	18.6 ± 6.7	28.2 ± 6.6	16.3 ± 4.1	< 0.001
**Intra-aortic balloon pump used**	4.6% (45)	19.9% (38)	0.9% (7)	< 0.001
**Number of vessels involved**				
Single vessel disease	36.7% (359)	31.4% (60)	38% (299)	0.002
Two vessel disease	32.3% (316)	27.2% (52)	33.6% (264)	
Three vessel disease	30.9% (302)	41.4% (79)	28.4% (223)	
**Culprit coronary artery**				
Left main	1.6% (16)	5.8% (11)	0.6% (5)	< 0.001
LAD: Proximal	34.3% (335)	49.7% (95)	30.5% (240)	
LAD: Non-Proximal	17% (166)	15.7% (30)	17.3% (136)	
Left circumflex	11.6% (113)	8.4% (16)	12.3% (97)	
Right coronary artery	34.2% (334)	19.9% (38)	37.7% (296)	
Diagonal	1% (10)	0% (0)	1.3% (10)	
Ramus	0.3% (3)	0.5% (1)	0.3% (2)	
**Pre-procedure TIMI (Thrombolysis in Myocardial Infarction) flow**				
0	56.1% (548)	71.7% (137)	52.3% (411)	< 0.001
I	18.5% (181)	15.7% (30)	19.2% (151)	
II	16.3% (159)	11% (21)	17.6% (138)	
III	9.1% (89)	1.6% (3)	10.9% (86)	
**Thrombus Grade**				
G1	4.1% (40)	0% (0)	5.1% (40)	< 0.001
G2	5% (49)	1.6% (3)	5.9% (46)	
G3	24.2% (236)	16.2% (31)	26.1% (205)	
G4	11.6% (113)	11.5% (22)	11.6% (91)	
G5	55.2% (539)	70.7% (135)	51.4% (404)	
**Mean vessel diameter (mm)**	3.5 ± 0.3	3.5 ± 0.3	3.5 ± 0.3	0.154
**Total lesion length (mm)**	27.6 ± 11.8	28.4 ± 13.6	27.5 ± 11.3	0.347
**Post-procedure TIMI (Thrombolysis in Myocardial Infarction) flow**				
0	0.8% (8)	1.6% (3)	0.6% (5)	< 0.001
I	2.5% (24)	7.3% (14)	1.3% (10)	
II	7.3% (71)	14.7% (28)	5.5% (43)	
III	89.5% (874)	76.4% (146)	92.6% (728)	
**In-hospital mortality**	4.3% (42)	14.1% (27)	1.9% (15)	< 0.001
**Follow-up**				
Successful follow-up	95.7% (935)	100% (191)	94.7% (744)	0.001
Follow-up duration (months)	5.9 ± 2.4	5.2 ± 3	6.1 ± 2.1	< 0.001
All-cause mortality	15% (140)	35.1% (67)	9.8% (73)	< 0.001
Stroke/CVA	0.9% (8)	0% (0)	1.1% (8)	0.371
Hospitalization due to HF	3.5% (33)	7.3% (14)	2.6% (19)	0.001
MI requiring revascularization	6.6% (62)	8.9% (17)	6% (45)	0.158
MACE	20% (187)	39.8% (76)	14.9% (111)	< 0.001

SBP = systolic blood pressure, LVEDP = left ventricular end-diastolic pressure, LAD = left anterior descending artery, CVA = cerebrovascular accidents, TIA = transient ischemic attack, HF = heart failure, MI = myocardial infarction

###  Post-procedure outcomes

 The in-hospital mortality rate was 4.3% (42) and after a mean follow-up of 5.9 ± 2.4 months, short-term all-cause mortality rate was 15% (140). Both in-hospital (14.1% vs. 1.9%; *P* < 0.001) and short-term mortality rate (35.1% vs. 9.8%; *P* < 0.001) was found to be significantly higher for patients with SBP/LVED *P* ≤ 5.4 compared to those with SBP/LVEDP > 5.4 (Table 2).

###  Receiver operating characteristics curve analysis

**Figure 1:**
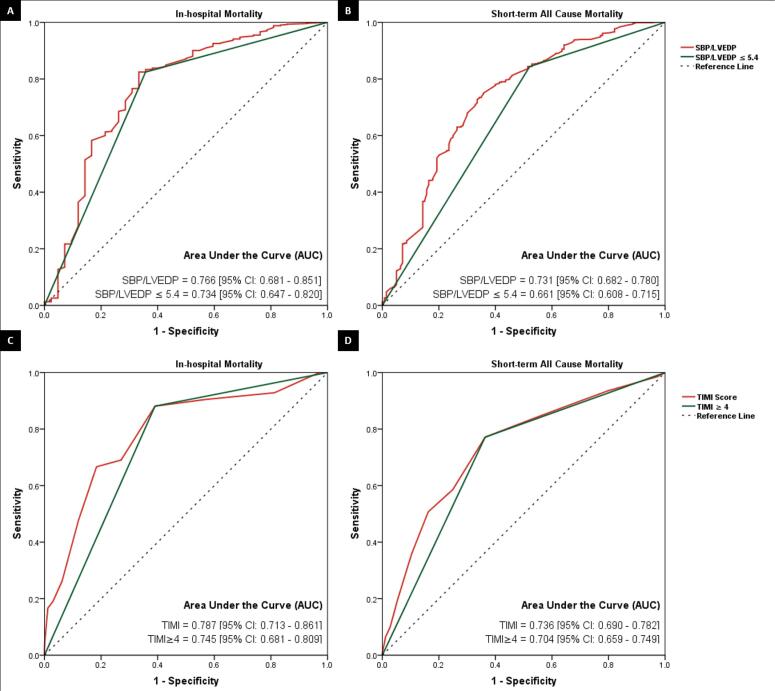
The receiver operating characteristics curve analysis of SBP to LVEDP ration and TIMI score for the prediction of in-hospital and short-term mortality after primary PCI SBP = systolic blood pressure, LVEDP = left ventricular end-diastolic pressure, TIMI = thrombolysis in myocardial infarction, CI = confidence interval

 The sensitivity and specificity analysis of SBP/LVEDP and TIMI score for the prediction of in-hospital mortality are presented in Table 3. The AUCs of SBP/LVEDP for the prediction of in-hospital and short-term mortality were 0.766 [95% CI: 0.681 – 0.851] and 0.731 [95% CI: 0.682 – 0.780], respectively. Similarly, the AUCs of TIMI score for the prediction of in-hospital and short-term mortality were 0.787 [95% CI: 0.713 – 0.861] and 0.736 [95% CI: 0.690 – 0.782], respectively (Figure 1).

**Table 3:** Accuracy analysis of SBP to LVEDP ratio and TIMI score for the prediction of in-hospital mortality after primary PCI

	**SBP/LVEDP≤5.4**	**TIMI Score≥4**
Sensitivity	64.3% [95% CI; 48.0% to 78.5%]	99.8% [95% CI; 99.6% to 99.9%]
Specificity	82.5% [95% CI; 79.9% to 84.6%]	61.0% [95% CI; 57.5% to 64.1%]
Positive Predictive Value	14.1% [95% CI; 11.2% to 17.7%]	88.2% [95% CI; 87.4% to 89.0%]
Negative Predictive Value	98.1% [95% CI; 97.2% to 98.7%]	99.1% [95% CI; 97.9% to 99.6%]
Accuracy	64.3% [95% CI; 48.0% to 78.5%]	99.8% [95% CI; 99.6% to 99.9%]

SBP = systolic blood pressure, LVEDP = left ventricular end-diastolic pressure, TIMI = thrombolysis in myocardial infarction

## Discussion

 Given the clinical significance of early risk stratification in STEMI patients, numerous risk stratification models have been formulated and validated over time.^10-13^ These models primarily aim to provide early warning signs to attending physicians regarding potential adverse events. Nevertheless, the computational complexity and reliance on non-routine parameters pose challenges to the practical applicability of most of these scoring systems.^14^ Therefore, this study aimed to assess the prognostic significance of a simple index based on the ratio of SBP to LVEDP. Our findings revealed that a low SBP/LVEDP ratio ( ≤ 5.4) is associated with an increased incidence of in-hospital (14.1% vs. 1.9%; *P* < 0.001) and short-term mortality (35.1% vs. 9.8%; *P* < 0.001) following primary PCI in a contemporary cohort of STEMI patients. The predictive performance of this simple index was found to be comparable to that of the TIMI risk score. The area under the curve (AUC) was 0.766 (95% CI: 0.681 - 0.851) for the prediction of in-hospital mortality and 0.731 (95% CI: 0.682 - 0.780) for the prediction of short-term all-cause mortality, while the AUC for the TIMI risk score was 0.787 (95% CI: 0.713 - 0.861) and 0.736 (95% CI: 0.690 - 0.782), respectively.

 Furthermore, during a mean follow-up period of 5.9 ± 2.4 months, patients with an SBP/LVEDP ratio ≤ 5.4 exhibited a significantly higher rate of major adverse cardiac events (MACE) compared to those with an SBP/LVEDP ratio > 5.4 (39.8% vs. 14.9%; *P* < 0.001). The low SBP to LVEDP ratio was associated with several factors, including longer ischemic time, higher heart rate, a greater incidence of anterior wall myocardial infarction, a higher prevalence of Killip class III/IV, an increased occurrence of arrhythmias and cardiac arrest, a higher prevalence of diabetes, involvement of three vessels, pre-procedure TIMI 0 flow, low post-procedure TIMI III flow, and a greater need for intra-aortic balloon pump (IABP) placement. These factors collectively contribute to the higher incidence of MACE observed in these patients.

 The only study thus far regarding the role of SBP to LVEDP ratio is conducted by Sola M et al.^14^ This study also reported similar predictive role of SBP/LVEDP for the prediction of in-hospital and 30-day mortality. The likelihood ratio for in-hospital death and IABP usage at SBP/LVEDP ratio of ≤ 4 was 4.7 and 5.8, respectively.^14^ Even though, clinical data regarding the prognostic strength of this simple ratio is very limited but prognostic significance of SBP and LVEDP has been extensively studied and reported in numerous clinical investigations.^19^ The prognostic role of SBP in patients with STEMI has been extensively established, making it a valuable prognostic indicator for various clinical outcomes.^15-17,19,20^ In clinical practice, SBP is commonly utilized in risk stratification models, such as the TIMI risk score, to assess the likelihood of future cardiovascular events and guide appropriate management strategies.^18^

 Considering its prognostic significance, several clinical indices have been developed based on SBP. These include the Shock Index (SI), Age-adjusted SI, Modified SI (MSI), TIMI risk index (TRI), and LASH score.^7,15-17,19,20^ Each of these indices takes into account SBP and utilizes it as a key component to predict patient outcomes and aid in risk stratification.

 Furthermore, left ventricular end-diastolic pressure (LVEDP) is another important invasive measure used to evaluate the hemodynamic status of patients. It has been identified as an independent and combined indicator of poor prognosis, in conjunction with other clinical parameters.^21-24^ By assessing LVEDP, healthcare professionals can gather valuable information regarding the patient’s cardiac function and prognosis. In summary, the prognostic role of SBP in STEMI patients has been well-established, and it is widely utilized in risk stratification models. Additionally, LVEDP serves as an important indicator of prognosis, both independently and in combination with other clinical parameters. These factors contribute to a comprehensive understanding of patient outcomes and assist in guiding appropriate clinical management decisions. After proper zeroing and calibration of Physiomonitoring system, as per standard protocol, simultaneous single invasive measurement of aortic and LVEDP has lesser measurement biasness as compared to any non-invasive alternative. Secondly, these two measures are routinely performed in our catheterization laboratory. Hence, better accuracy and reliability of these readily available measures makes SBP to LVEDP ratio an attractive choice for risk categorization of these patients. While LVEDP is indeed an invasive marker, its routine measurement during primary PCI and simplicity of the calculation, makes SBP to LVEDP ratio an attractive alternative to complex clinical scoring systems like TIMI, PAMI, GRACE, and CADILLAC scores. Moreover, the invasive nature of LVEDP assessment improves its reliability and positions it as a preferable tool in risk stratification for STEMI patients undergoing intervention.

 This observational study utilized prospectively collected data from an adequate number of patients. However, it is important to note that the main limitation of this study is its single-center coverage, which may impact the generalizability of the findings. Additionally, it should be acknowledged that invasive measures such as LVEDP may exhibit inter-operator variability, which could influence the results.

 To establish a more robust and comprehensive risk stratification index for patients with STEMI, further large-scale multicenter prospective studies are necessary. These studies would help validate the findings of this study and provide a more reliable and widely applicable risk assessment tool for STEMI patients.

## Conclusion

 In conclusion, the ratio of SBP to LVEDP (SBP/LVEDP) demonstrated a strong predictive ability, comparable to the TIMI risk score. SBP/LVEDP is a readily available ratio that can rapidly provide valuable prognostic information during primary PCI. With the help of this useful ratio at the time of procedure, it is quite possible to identify a subgroup of patients with a heightened risk of both in-hospital and short-term mortality. This can offer an opportunity to implement more cautious and aggressive management strategies in the Cardiac Catheterization Laboratory, potentially leading to improved outcomes for these high-risk individuals.

## Acknowledgments

 The authors wish to acknowledge the support of the staff members of the Clinical Research Department of the National Institute of Cardiovascular Diseases (NICVD) Karachi, Pakistan.

## Competing Interests

 The authors declare that they have no conflicts of interest.

## Ethical Approval

 This study was approval by the ethical review committee of the National Institute of Cardiovascular Diseases (NICVD), Karachi (ERC-30/2020).

## References

[1] Krumholz HM, Normand ST, Wang Y (2019). Twenty-year trends in outcomes for older adults with acute myocardial infarction in the United States. JAMA Netw Open.

[2] Kumar R, Shah JA, Solangi BA, Ammar A, Kumar M, Khan N (2022). The burden of short-term major adverse cardiac events and its determinants after emergency percutaneous coronary revascularization: a prospective follow-up study. J Saudi Heart Assoc.

[3] McManus DD, Gore J, Yarzebski J, Spencer F, Lessard D, Goldberg RJ (2011). Recent trends in the incidence, treatment, and outcomes of patients with STEMI and NSTEMI. Am J Med.

[4] Taniwaki M, Stefanini GG, Räber L, Brugaletta S, Cequier A, Heg D (2015). Predictors of adverse events among patients undergoing primary percutaneous coronary intervention: insights from a pooled analysis of the COMFORTABLE AMI and EXAMINATION trials. EuroIntervention.

[5] Guo Y, Yin F, Fan C, Wang Z (2018). Gender difference in clinical outcomes of the patients with coronary artery disease after percutaneous coronary intervention: a systematic review and meta-analysis. Medicine (Baltimore).

[6] Kong S, Chen C, Zheng G, Yao H, Li J, Ye H (2021). A prognostic nomogram for long-term major adverse cardiovascular events in patients with acute coronary syndrome after percutaneous coronary intervention. BMC Cardiovasc Disord.

[7] Kumar R, Ahmed I, Rai L, Khowaja S, Hashim M, Huma Z (2022). Comparative analysis of four established risk scores for prediction of in-hospital mortality in patients undergoing primary percutaneous coronary intervention. Am J Cardiovasc Dis.

[8] Shangguan Q, Xu JS, Su H, Li JX, Wang WY, Hong K (2015). Modified shock index is a predictor for 7-day outcomes in patients with STEMI. Am J Emerg Med.

[9] Abreu G, Azevedo P, Galvão Braga C, Vieira C, Álvares Pereira M, Martins J (2018). Modified shock index: a bedside clinical index for risk assessment of ST-segment elevation myocardial infarction at presentation. Rev Port Cardiol (Engl Ed).

[10] Morrow DA, Antman EM, Parsons L, de Lemos JA, Cannon CP, Giugliano RP (2001). Application of the TIMI risk score for ST-elevation MI in the National Registry of Myocardial Infarction 3. JAMA.

[11] Addala S, Grines CL, Dixon SR, Stone GW, Boura JA, Ochoa AB (2004). Predicting mortality in patients with ST-elevation myocardial infarction treated with primary percutaneous coronary intervention (PAMI risk score). Am J Cardiol.

[12] Granger CB, Goldberg RJ, Dabbous O, Pieper KS, Eagle KA, Cannon CP (2003). Predictors of hospital mortality in the global registry of acute coronary events. Arch Intern Med.

[13] Halkin A, Singh M, Nikolsky E, Grines CL, Tcheng JE, Garcia E (2005). Prediction of mortality after primary percutaneous coronary intervention for acute myocardial infarction: the CADILLAC risk score. J Am Coll Cardiol.

[14] Sola M, Venkatesh K, Caughey M, Rayson R, Dai X, Stouffer GA (2017). Ratio of systolic blood pressure to left ventricular end-diastolic pressure at the time of primary percutaneous coronary intervention predicts in-hospital mortality in patients with ST-elevation myocardial infarction. Catheter Cardiovasc Interv.

[15] Wang G, Wang R, Liu L, Wang J, Zhou L (2021). Comparison of shock index-based risk indices for predicting in-hospital outcomes in patients with ST-segment elevation myocardial infarction undergoing percutaneous coronary intervention. J Int Med Res.

[16] Zhou J, Shan PR, Xie QL, Zhou XD, Cai MX, Xu TC (2019). Age shock index and age-modified shock index are strong predictors of outcomes in ST-segment elevation myocardial infarction patients undergoing emergency percutaneous coronary intervention. Coron Artery Dis.

[17] Zhang X, Wang Z, Wang Z, Fang M, Shu Z (2017). The prognostic value of shock index for the outcomes of acute myocardial infarction patients: a systematic review and meta-analysis. Medicine (Baltimore).

[18] Morrow DA, Antman EM, Charlesworth A, Cairns R, Murphy SA, de Lemos JA (2000). TIMI risk score for ST-elevation myocardial infarction: a convenient, bedside, clinical score for risk assessment at presentation: an intravenous nPA for treatment of infarcting myocardium early II trial substudy. Circulation.

[19] Warren J, Nanayakkara S, Andrianopoulos N, Brennan A, Dinh D, Yudi M (2019). Impact of pre-procedural blood pressure on long-term outcomes following percutaneous coronary intervention. J Am Coll Cardiol.

[20] Abreu G, Azevedo P, Galvão Braga C, Vieira C, Álvares Pereira M, Martins J (2018). Modified shock index: a bedside clinical index for risk assessment of ST-segment elevation myocardial infarction at presentation. Rev Port Cardiol (Engl Ed).

[21] Azzalini L, Seth M, Sukul D, Arora DS, Chattahi J, Osman A (2022). Impact of left ventricular end-diastolic pressure on the outcomes of patients undergoing percutaneous coronary intervention. Am J Cardiol.

[22] Araujo GN, Beltrame R, Pinheiro Machado G, Luchese Custodio J, Zimerman A, Donelli da Silveira A (2021). Comparison of admission lung ultrasound and left ventricular end-diastolic pressure in patients undergoing primary percutaneous coronary intervention. Circ Cardiovasc Imaging.

[23] Ammar A, Khowaja S, Kumar R, Kumar V, Hussain A, Ahmed S (2022). Significance of left ventricular end diastolic pressure for risk stratification of contrast-induced acute kidney injury after primary percutaneous coronary intervention. Pak Heart J.

[24] Ndrepepa G, Cassese S, Emmer M, Mayer K, Kufner S, Xhepa E (2019). Relation of ratio of left ventricular ejection fraction to left ventricular end-diastolic pressure to long-term prognosis after ST-segment elevation acute myocardial infarction. Am J Cardiol.

